# Magnetic-field-enhanced laser-induced electron coherence in 2D magnetic materials

**DOI:** 10.1093/nsr/nwag594

**Published:** 2026-09-12

**Authors:** Yixuan Huang, Sheng Meng, Jimin Zhao

**Affiliations:** Beijing National Laboratory for Condensed Matter Physics, Institute of Physics, Chinese Academy of Sciences, Beijing 100190, China; School of Physical Sciences, University of Chinese Academy of Sciences, Beijing 100049, China; Beijing National Laboratory for Condensed Matter Physics, Institute of Physics, Chinese Academy of Sciences, Beijing 100190, China; School of Physical Sciences, University of Chinese Academy of Sciences, Beijing 100049, China; Songshan Lake Materials Laboratory, Dongguan 523808, China; Beijing National Laboratory for Condensed Matter Physics, Institute of Physics, Chinese Academy of Sciences, Beijing 100190, China; School of Physical Sciences, University of Chinese Academy of Sciences, Beijing 100049, China; Songshan Lake Materials Laboratory, Dongguan 523808, China

**Keywords:** non-linear optics, laser-induced, light-matter interactions, magnetic materials, all-optical switch

## Abstract

Laser-induced electron coherence is a fascinating behavior in quantum materials. However, to date, viable approaches to control this excited-state behavior remain lacking. Here, we overturn this by realizing magnetic-field-controlled spatial self-phase modulation (SSPM), a third-order non-linear optical response. Light-matter interactions in ferromagnetic VSe_2_ and diamagnetic graphene are observed to be precisely controlled by an external magnetic field, achieving an intriguing anisotropic SSPM, accompanied by prominent optical birefringence. We attribute this magnetic-field-controlled SSPM to the magnetic-field-induced broken symmetry in the collective behavior of flakes. Significantly, the non-linear optical coefficient *χ*^(3)^ is enhanced by 58%, which breaks the record of largest *χ*^(3)^. Furthermore, we demonstrate a magnetic-field-controlled all-optical switch based on SSPM in VSe_2_, achieving an enhancement of 340% in the signal-to-control intensity ratio (manifesting the weak-light-control-strong-light capability). Our findings usher in a new paradigm, controlling (rather than merely monitoring) the laser-induced electron coherence in SSPM, which enables the next generation of all-optical switching based on SSPM.

## Teaser

Magnetic field are shown to be able to control a third-order nonlinear optical response in magnetic 2D materials, which modifies the laser-induced electron coherence and enables a boosted all-optical switch.

## INTRODUCTION

Electron coherence is the underlying pivotal factor for various intriguing quantum states and emergent phenomena in quantum materials [1–3]. However, unavoidable fluctuations pose significant challenges to achieving spontaneous electron coherence [4]. Thus, achieving laser-controlled electron coherence is one of the cutting-edge frontiers, which has been exemplified in extensive coherent light-matter interactions, such as Dicke cooperativity [2] and Floquet states [5]. Through coherent light-matter interactions, switching of optical non-linearity has been achieved [5], which holds potential for applications.

Among these, spatial self-phase modulation (SSPM) [1,6,7], a third-order non-linear optical response [8], has been attributed to laser-induced electron coherence [1] and widely investigated in two-dimensional (2D) quantum materials [1,6,9–20]. The synchronized (i.e. coherent) electronic oscillation is driven by the optical field and can be described by the anharmonic Lorentz oscillator model [21,22], leading to a non-linear optical phase and thus diffractive fringes in the far field [8]. Furthermore, an all-optical switch based on SSPM has been demonstrated [1], which realized the capability of using a weak light to control a strong light [23–27], resembling an electronic transistor where a weak current controls a strong current.

However, to date, all reported SSPM responses are intrinsic properties of quantum materials—there has been no report of viable ways to control or enhance the value of *χ*^(3)^. Despite of great efforts in searching materials with higher *χ*^(3)^ values, no breakthrough has been achieved and the *χ*^(3)^ value of graphene [6,7] has remained to be the world record [7]. Consequently, it is both challenging and valuable to achieve an enhanced value of *χ*^(3)^, hopefully higher than that of graphene.

In this work, we surpass all previous SSPM studies by demonstrating the first realization of field-controlled (enhanced) SSPM. The *χ*^(3)^ values are controlled and significantly enhanced, up to 58% in VSe_2_ and 32% in graphene. Significantly, we report the highest value of *χ*^(3)^ achieved so far, setting a new record. The magnetic field also induces an anisotropic SSPM, accompanied by an optical birefringence, which has never been observed before. Furthermore, we also demonstrate the first magnetic-field-controlled all-optical switch based on SSPM. A 340% enhancement in the signal-to-control intensity ratio has been detected. Our work ushers in a cutting-edge frontier for the SSPM investigation: controlling laser-induced electron coherence in quantum materials by external fields.

## RESULTS AND DISCUSSION

### Wavelength-dependent SSPM in VSe_2_

Experiments are carried out in dispersed suspensions of VSe_2_, a 2D room-temperature ferromagnetic material [28–30], and graphene, a 2D strongly diamagnetic material [31]. The non-linear optical response in VSe_2_ has not been extensively explored before [32]. Thus, we first study SSPM in VSe_2_ without external field, using lasers with various wavelengths. The experimental set-up is schematically shown in Fig. 1a (for detailed descriptions, see Materials and methods section). Typical SSPM patterns under different wavelengths are presented in Fig. 1b. The intensity distribution in the patterns is consistent with the calculation [8] result (insets of Fig. 1c), confirming the patterns emerge from SSPM.

**Figure 1.**
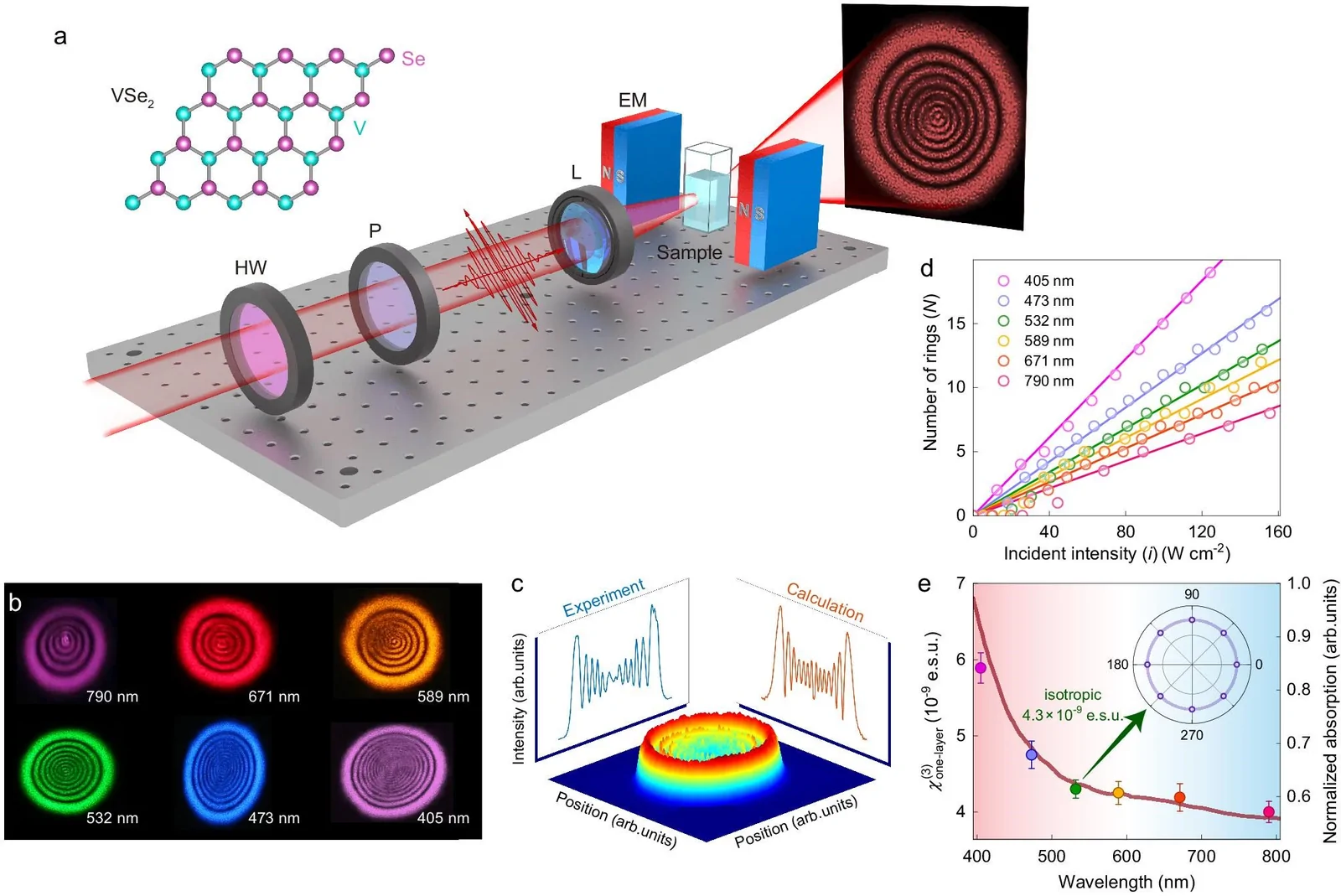
Schematic experimental set-up and wavelength-dependent SSPM in VSe_2_. (a) Schematic experimental set-up for magnetic-field-controlled SSPM. Laser beam traverses the sample, whereby the magnetic field is perpendicular to the light propagation. HW: half-wave plate. P: polarizer. L: focusing lens. EM: electromagnet. Inset: schematic lattice structure of 2*H*-VSe_2_. (b) Typical SSPM patterns from the VSe_2_ suspension excited by 100 W/cm^2^ laser beams at different wavelengths. (c) Intensity distribution of the 532 nm SSPM pattern. Curves: experimental and calculated results of radial distribution. (d) Linear *N*–*I* relations for the VSe_2_ suspension under varying laser wavelengths without external field. Solid lines: fitting lines for the data. (e) $\chi _{{\mathrm{one - layer}}}^{{\mathrm{(3)}}}$ values of VSe_2_ without external field, proportional to the absorbance (curve). Inset: isotropic *χ*^(3)^ at *B* = 0 T.

To obtain the *χ*^(3)^ values, we record the number of SSPM rings *N* and corresponding laser intensity *I* for each wavelength (Fig. 1d; for details of extracting *N* value, please see Supplementary data). The *N*–*I* relations exhibit linear dependences. By fitting the *N*–*I* relations (for detailed data analysis procedure and its limitation, please see Supplementary data), we obtain the *χ*^(3)^ values for the whole suspension and effective one-layer VSe_2_, denoted as $\chi _{{\mathrm{total}}}^{{\mathrm{(3)}}}$ and $\chi _{{\mathrm{one - layer}}}^{{\mathrm{(3)}}}$, respectively. We summarize the values for $\chi _{{\mathrm{one - layer}}}^{{\mathrm{(3)}}}$ in Fig. 1e, whereby VSe_2_ exhibits a broadband SSPM response in the visible to near-infrared light regime. The values of $\chi _{{\mathrm{one - layer}}}^{{\mathrm{(3)}}}$ are relatively large, which is negatively correlated with the laser wavelength and proportional to the absorbance of VSe_2_ (Fig. 1e). As expected, we experimentally confirm that *χ*^(3)^ is independent on the laser polarization (i.e. isotropic SSPM) in the absence of magnetic field (inset of Fig. 1e).

### Magnetic-field-controlled SSPM

We investigate the magnetic-field-controlled SSPM in the VSe_2_ sample. As shown in Fig. 1a, an external magnetic field is applied perpendicular to the laser beam propagating direction. We measure the *N*–*I* relations under different laser polarizations, marked by the angle *θ* (Fig. S1). For each value of *θ*, the $\chi _{{\mathrm{total}}}^{{\mathrm{(3)}}}$ value is obtained by fitting the *N*–*I* relation. For each magnetic field intensity *B*, the same procedure is performed and all obtained values are summarized in Fig. 2a. Under different *B*, we obtain different closed curves showing the dependence of $\chi _{{\mathrm{total}}}^{{\mathrm{(3)}}}$ on *θ*. Interestingly, a novel anisotropic SSPM behavior is observed. In Fig. 2a, the closed curves start from a perfect circle corresponding to the isotropic SSPM without external field, to change into ellipses for relatively weak *B* field, before finally transforming into peanut curves for relatively strong *B* field. Such a novel behavior has never been reported before.

**Figure 2.**
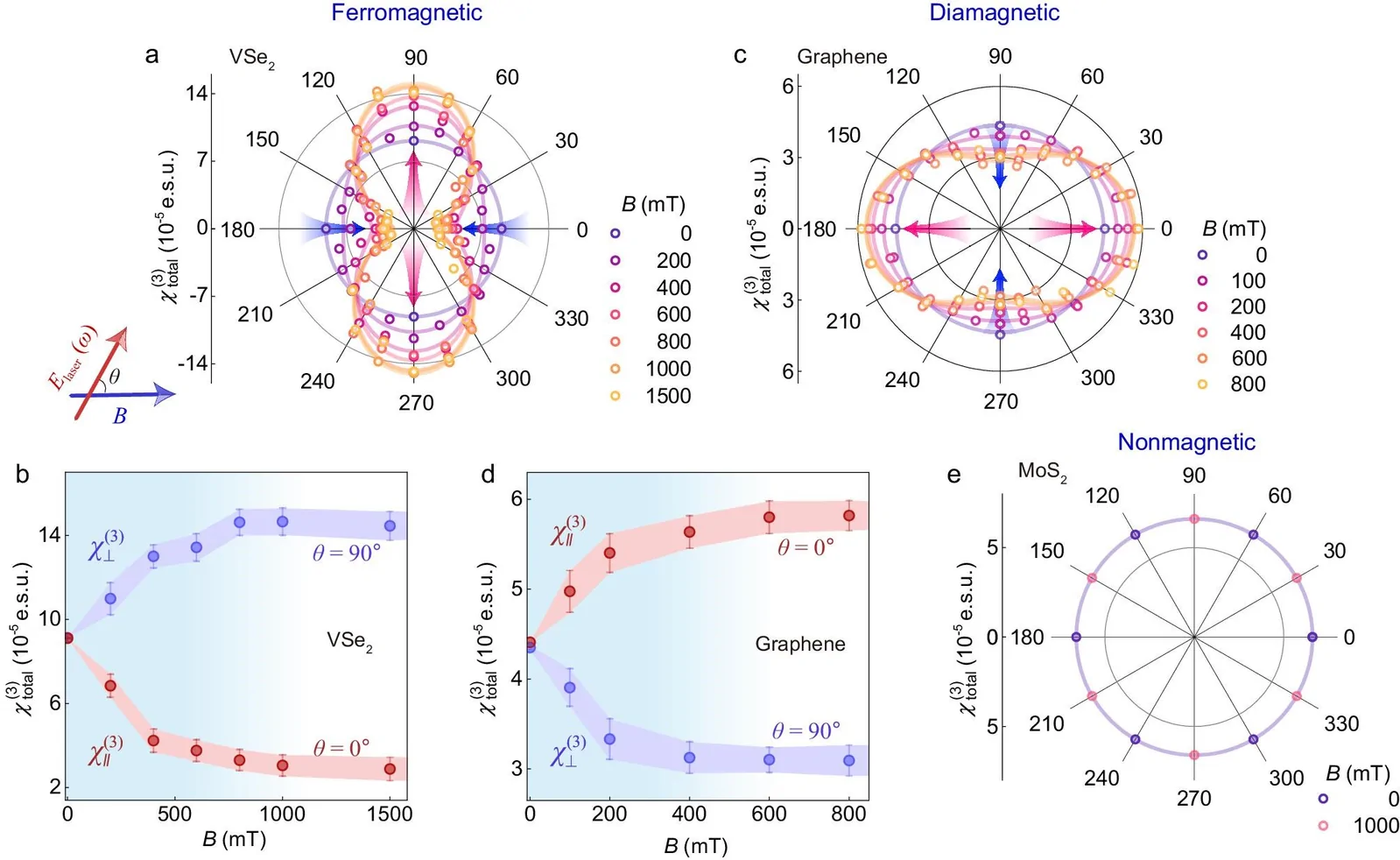
Magnetic-field-controlled SSPM in VSe_2_ and graphene suspensions. (a) Polarization dependences of $\chi _{{\mathrm{total}}}^{{\mathrm{(3)}}}$ for ferromagnetic VSe_2_ suspension under different *B* field intensity, revealing magnetic-field-controlled SSPM and anisotropic SSPM. (b) $\chi _\parallel ^{(3)}$ and $\chi _ \bot ^{(3)}$ values for the VSe_2_ suspension controlled by the *B* field. $\chi _ \bot ^{(3)}$: average of $\chi _{{\mathrm{total}}}^{{\mathrm{(3)}}}$ values for *θ* = 90° and 270°. $\chi _\parallel ^{(3)}$: average of $\chi _{{\mathrm{total}}}^{{\mathrm{(3)}}}$ values for *θ* = 0° and 180°. (c) Polarization dependences of $\chi _{{\mathrm{total}}}^{{\mathrm{(3)}}}$ for diamagnetic graphene suspension. (d) $\chi _\parallel ^{(3)}$ and $\chi _ \bot ^{(3)}$ values for graphene suspension controlled by the *B* field. (e) Polarization dependences of $\chi _{{\mathrm{total}}}^{{\mathrm{(3)}}}$ for non-magnetic MoS_2_ suspension. No magnetic-field-controlled SSPM is observed. All laser wavelengths are 532 nm.

These $\chi _{{\mathrm{total}}}^{{\mathrm{(3)}}}$ patterns exhibit one C_2_ rotational symmetry and two mirror symmetries (Fig. 2a). For each *B*, $\chi _{{\mathrm{total}}}^{{\mathrm{(3)}}}$ maximizes at *θ* = 90° and 270° (denoted as $\chi _ \bot ^{(3)}$) and minimizes at *θ* = 0° and 180° (denoted as $\chi _{\parallel} ^{(3)}$). We plot the $\chi _ \bot ^{(3)}$ and $\chi _\parallel ^{(3)}$ values under different *B* in Fig. 2b. For *B* = 0 mT, $\chi _ \bot ^{(3)}$=$\chi _\parallel ^{(3)}$= 9.1 × 10^−5^ e.s.u. As *B* increases, $\chi _ \bot ^{(3)}$ exhibits a 58% enhancement to become 1.44 × 10^−4^ e.s.u. at 1000 mT, and saturates at even higher *B*. On the contrary, $\chi _\parallel ^{(3)}$ exhibits a 70% decreasement to become 2.7 × 10^−5^ e.s.u. at 1000 mT. We record that the ratio $\chi _ \bot ^{(3)}/\chi _\parallel ^{(3)} = 1$ at *B* = 0 mT is enhanced to $\chi _ \bot ^{(3)}/\chi _\parallel ^{(3)} = 5.2$ at *B* = 1000 mT, unambiguously demonstrating that the anisotropy in SSPM is induced and enhanced by the magnetic field.

Similarly, we show that magnetic-field-controlled SSPM is also observed in diamagnetic [31] few-layer graphene suspension (Fig. 2c, see *N*–*I* relations in Fig. S2), which is also anisotropic. However, unlike that VSe_2_ exhibits a relation $\chi _\parallel ^{(3)} < \chi _ \bot ^{(3)}$, graphene manifests an opposite relation $\chi _\parallel ^{(3)} > \chi _ \bot ^{(3)}$. For each given *B*, $\chi _{{\mathrm{total}}}^{{\mathrm{(3)}}}$ of graphene maximizes at *θ* = 0° and 180° ($\chi _\parallel ^{(3)}$) and minimizes at *θ* = 90° and 270° ($\chi _ \bot ^{(3)}$). We plot the *B* field dependence of $\chi _ \bot ^{(3)}$ and $\chi _\parallel ^{(3)}$ in Fig. 2d. For *B* = 0 mT, $\chi _ \bot ^{(3)}$ = $\chi _\parallel ^{(3)}$ = 4.41 × 10^−5^ e.s.u. As *B* increases, $\chi _\parallel ^{(3)}$ exhibits a 32% enhancement to become 5.8 × 10^−5^ e.s.u. at 400 mT, saturating at higher *B* field; in contrast, $\chi _ \bot ^{(3)}$ exhibits a 30% decrease to become 3.1 × 10^−5^ e.s.u. at 400 mT. Consequently, $\chi _ \bot ^{(3)}/\chi _\parallel ^{(3)} = 1$ at *B* = 0 mT decreases to $\chi _ \bot ^{(3)}/\chi _\parallel ^{(3)} = 0.58$ at *B* = 400 mT.

Significantly, we break the record of the largest *χ*^(3)^ value [6,33] by obtaining a magnetic-field-enhanced value of $\chi _{{\mathrm{one - layer}}}^{{\mathrm{(3)}}}$= 2.0 × 10^−7^ e.s.u., for graphene at *B* = 600 mT. We verify this by obtaining an identical value of $\chi _{{\mathrm{one - layer}}}^{{\mathrm{(3)}}}$= 1.5 × 10^−7^ e.s.u. at *B* = 0 mT (see Materials and methods section) as previous report [6,33]. Furthermore, we unambiguously demonstrate the magnetic-field-controlled SSPM is absent in a suspension of non-magnetic [34] MoS_2_ flakes (Fig. 2e).

### Magnetic-field-induced optical birefringence

We further investigate the magnetic-field-induced asymmetry in light propagation. Specifically, we find magnetic-field-induced optical birefringence in our experiment. The light polarization of the outcoming beam, which are composed of concentric nested cones forming the pattern on far field screen [1,6,7], is monitored. Given the fixed direction of the *B*, the ordinary light polarization is along *θ* = 0° (along the magnetic field) and the extraordinary light polarization is along *θ* = 90° (see Supplementary data). We tune the incident beam polarization to be *θ* = 45° to have equal intensity for the ordinary and extraordinary lights. A polarizer is placed behind the sample, of which we tune the azimuth angle *Ф* and record the transmitted intensity, for each *B* field intensity.

The results for VSe_2_ and graphene suspensions are presented in Fig. 3a and b, respectively. At *B* = 0, the intensity after the polarizer exhibits a maximum at *θ* = 45° and a minimum (i.e., 0) at *θ* = 135°. The figure-eight-shaped intensity distribution is consistent with a linearly polarized outcoming laser beam (for detailed explanation, see Materials and methods section). There is no birefringence in this case. With *B* increasing, the recorded intensity distribution changes from a figure-eight shape to a peanut shape, which manifests that the outcoming laser beam becomes elliptically polarized. This elliptical polarization is due to the birefringence induced by the magnetic field. Finally, the intensity distribution becomes a perfect ring for a large *B* field, which manifests a circularly polarized outcoming laser beam. Further increasing the *B* field intensity results in saturation—that the shape of circle does not change anymore. The overall behavior reveals an unambiguous magnetic-field-induced (and -controlled) birefringence in the suspensions, which has not been reported in SSPM before.

**Figure 3.**
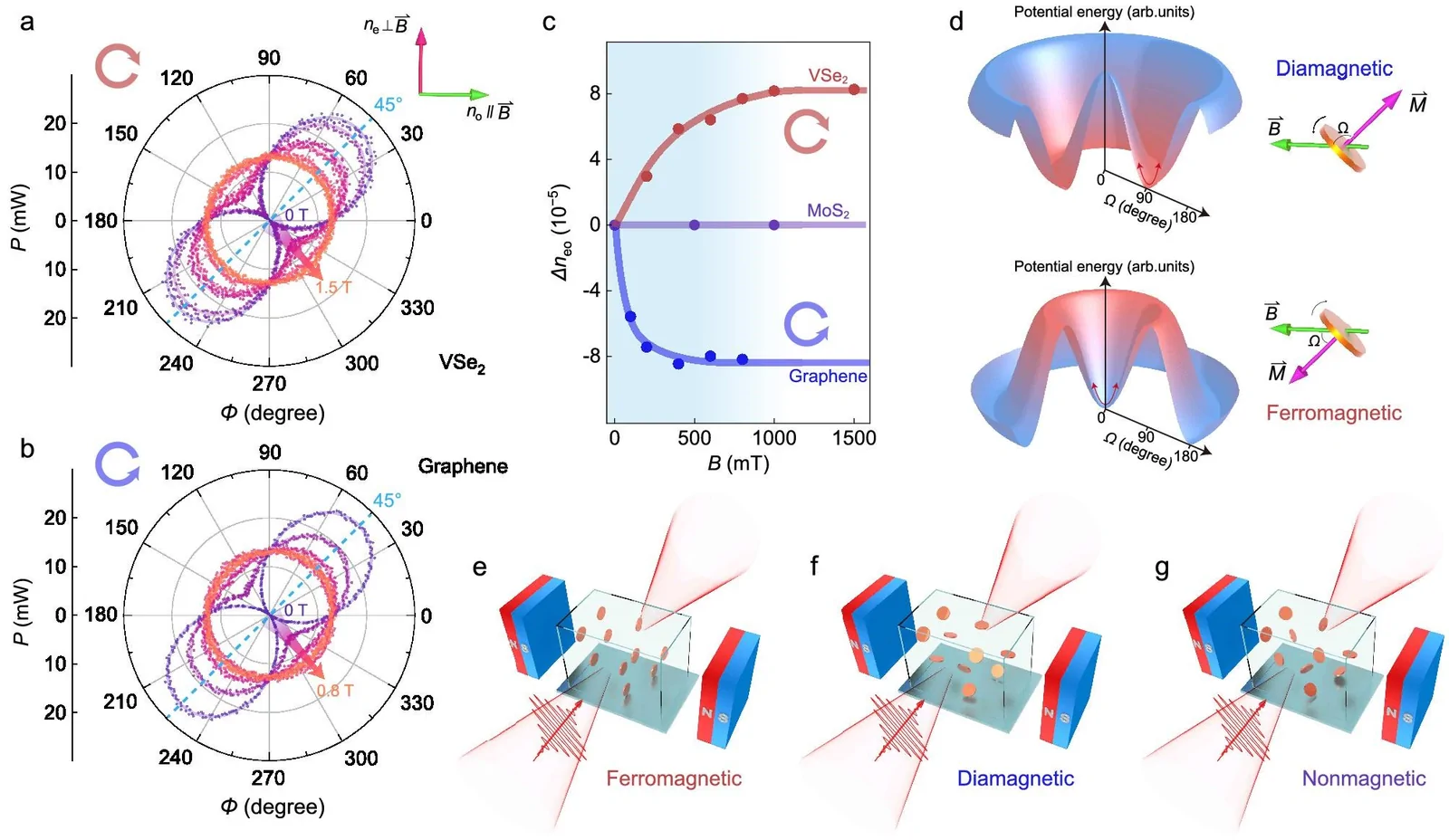
Magnetic-field-induced optical birefringence and microscopic illustration of flake alignment. (a) Polarization distribution of the transmitted light from VSe_2_ suspension, controlled by the magnetic-field-induced optical birefringence. The transmitted light is right-handed polarized. The polarization varies from linear polarization (figure-eight-shaped) without *B* field to elliptical polarization (peanut-shaped) at weak *B* field and finally to circular polarization (ring-shaped) at strong *B* field. Inset: polarization direction of the ordinary and extraordinary lights. (b) Polarization distribution of the transmitted light from graphene suspension. The transmitted light is left-handed polarized. (c) Difference in refractive indices Δ*n*_eo_ = *n*_e_ − *n*_o_, as controlled by the magnetic field. (d) Potential energies and magnetizations (inset) for ferromagnetic VSe_2_ and diamagnetic graphene. (e–g) Microscopic illustration of magnetic-field-controlled flake alignment for three types of samples with different magnetisms. The flakes are aligned perpendicular to the *B* field in panel (e), parallel to the *B* field in panel (f) and randomly in panel (g).

We further quantify the optical birefringence for the suspensions. By fitting the data in Fig. 3a and b (see Materials and methods section), we obtain difference between the refractive indices for ordinary light *n*_o_ and extraordinary light *n*_e_, denoted as Δ*n*_eo_ = *n*_e_ − *n*_o_ (Fig. 3c). The birefringence of both VSe_2_ and graphene increases with *B* and further saturates. Intriguingly, the transmitted light from VSe_2_ suspension is right-handed polarized, while that for graphene suspension is left-handed polarized (Fig. 3a and b, see Materials and methods section). Consequently, VSe_2_ exhibits positive Δ*n*_eo_ as a positive uniaxial crystal (see Supplementary data), while graphene manifests negative Δ*n*_eo_ as a negative uniaxial crystal. In addition, the magnetic-field-induced optical birefringence is absent in non-magnetic [34] MoS_2_, manifested by Δ*n*_eo_ = 0 in Fig. 3c.

### Mechanism of magnetic-field-controlled SSPM

Furthermore, we immobilize graphene and VSe_2_ flakes in epoxy resin, in which magnetic field control of SSPM and optical birefringence are found to be absent (see Supplementary data). This result suggests that the magnetic field aligns the nanoflakes in a collective way, which modifies the SSPM response. When there is no magnetic field, the flakes are randomly oriented. When there is a magnetic field, for magnetic materials, the field magnetizes and reorients the flakes. The magnetic moments of the thin flakes are out-of-plane [30,31] as $m = {{{\chi }_MB{\mathrm{cos}}(\Omega )} {/ {\vphantom {{{\chi }_MB{\mathrm{cos}}(\Omega )} {{\mu }_0}}} } {{\mu }_0}}$ (per unit volume), where Ω is the angle between the flakes’ magnetic moment (along the *c* axis [30,31]) and the external field, ${\chi }_M$ is the magnetic susceptibility and *μ*_0_ is the vacuum permeability. Thus, the potential energy introduced by the field is $U(\Omega )\ = - {{{\chi }_M{B}^2{\mathrm{co}}{{\mathrm{s}}}^2(\Omega )} {/ {\vphantom {{{\chi }_M{B}^2{\mathrm{co}}{{\mathrm{s}}}^2(\Omega )} {{\mu }_0}}} } {{\mu }_0}}$.

In Fig. 3d, we schematically plot the potential energy, which are in Mexican hat shapes. Notably, the ferromagnetic and diamagnetic flakes exhibit opposite shapes of potential energy, because ${\chi }_M$ > 0 in ferromagnetic VSe_2_ and ${\chi }_M$ < 0 in diamagnetic graphene (Fig. 3d). The different behaviors could be understood as follows. With the external magnetic field, the associated potential energy *U*(Ω) assumes its minimum value after the field rotates the flakes, reaching a steady state. We schematically illustrate the microscopic picture in Fig. 3e–g. For ferromagnetic VSe_2_ flakes, they are aligned so that Ω = 0°, with *c* axis being along the magnetic field (Fig. 3e); for diamagnetic graphene, the flakes are aligned such that Ω = 90°, with the *ab*-plane being along the magnetic field (Fig. 3f). Moreover, for non-magnetic [34] MoS_2_, owing to the negligible value of ${\chi }_M$, the flakes are insensitive to the magnetic field and assume a random orientation (Fig. 3g). Overall, the magnetic field primarily induces collective orientational alignment of anisotropic flakes in the suspension. The intrinsic anisotropic non-linear susceptibility of the individual flakes already exists in the absence of the magnetic field, while the field tunes orientational averaging and reveals larger tensor components of the non-linear response macroscopically. The flake orientation is also evidenced by the viscosity effect, where the time of flake alignment, probed by the optical birefringence, positively correlates to the solution viscosity (see Supplementary data).

The microscopic picture of flake alignment (Fig. 3e–g) can explain the observation of magnetic-field-induced optical birefringence in our experiment. Thin 2D materials exhibit a large in-plane refractive index and a relatively small out-of-plane refractive index. The alignment of flakes (Fig. 3e and f) leads to asymmetric refractive indices, which manifest as optical birefringences. Explicitly, for aligned ferromagnetic VSe_2_ flakes (Fig. 3e), the *ab*-plane is along the extraordinary axis (⊥*B*), leading to *n*_e_ > *n*_o_, resulting in the observed right-handed elliptical or circular polarized light (Fig. 3a); for aligned diamagnetic graphene flakes (Fig. 3f), the *ab*-plane is along the ordinary axis (∥*B*), leading to *n*_o_ > *n*_e_, resulting in the observed left-handed elliptical or circular polarized light (Fig. 3b); for randomly oriented non-magnetic MoS_2_ flakes (Fig. 3g), the refractive indices are isotropic, leading to no birefringence, resulting in only linearly polarized light (Fig. 3c). Furthermore, the optical birefringence in SSPM does not support the thermal lensing explanation for SSPM, where non-linear optical response is attributed to the laser-heated solvent. Because the refractive index of solvent is isotropic, the transmitted laser beam forming the SSPM pattern tends to have linear polarization, which is against our observation in this experiment.

Magnetic field control of SSPM is a field-controlled collective behavior in separated systems of 2D materials. The microscopic picture of flake alignment (Fig. 3e–g) can be used to explain the experimental results in Fig. 2a and c. In Fig. 3e, the VSe_2_ flakes are aligned perpendicular to the *B* field. When the laser beam is polarized along *θ* = 90°, the electric field of the light wave is in-plane, which can drive the carriers to move freely, leading to a large $\chi _{{\mathrm{total}}}^{{\mathrm{(3)}}}$ at *θ* = 90° (Fig. 2a). As a comparison, when the laser beam polarization is at *θ* = 0°, the electric field is out-of-plane, which can hardly drive the carriers to move freely, leading to a small $\chi _{{\mathrm{total}}}^{{\mathrm{(3)}}}$ at *θ* = 0° (Fig. 2a). In parallel, in Fig. 3f, the graphene flakes are aligned parallel to the *B* field. When the laser beam is polarized at *θ* = 0°, the electric field of the light wave is in-plane, which can drive the carriers to move freely, leading to a large $\chi _{{\mathrm{total}}}^{{\mathrm{(3)}}}$ at *θ* = 0° (Fig. 2c). When *θ* = 90°, because the flakes preserve a degree of freedom by rotating along the magnetic field (Fig. 3f), the electric field has both in-plane and out-of-plane components, leading to a smaller but moderate value of $\chi _{{\mathrm{total}}}^{{\mathrm{(3)}}}$ at *θ* = 90° (Fig. 2c).

Note that not all flakes are well-aligned due to insufficient *B* field intensity and demagnetization in thicker flakes [29]. Those randomly oriented flakes contribute an isotropic term in $\chi _{{\mathrm{total}}}^{{\mathrm{(3)}}}$. Denoting the number of effective layers of well-aligned flakes as *M*_1_ and non-aligned flakes as *M*_2_, *M*_1_ and *M*_2_ are controlled by the *B* field. Quantitatively, for VSe_2_ suspension, we derive (see Supplementary data)


(1) \begin{eqnarray*}
\chi _{{\mathrm{total}}}^{{\mathrm{(3)}}}(B,\theta ) &=& ( {M}_1{{(B)}}^2{{\sin }}^2(\theta )\\
&& + {M}_2{{(B)}}^2)\chi _{{\mathrm{one - layer}}}^{{\mathrm{(3)}}}.\end{eqnarray*}

For graphene suspension, we derive


(2) \begin{eqnarray*}\chi _{{\mathrm{total}}}^{{\mathrm{(3)}}}(B,\theta ) &=& \bigg( {{M}_1{{(B)}}^2\bigg( {1 - \frac{1}{2}{{\sin }}^2( \theta )} \bigg) }\\
&&+\, {{M}_2{{(B)}}^2} \bigg)\chi _{{\mathrm{one - layer}}}^{{\mathrm{(3)}}}.
\end{eqnarray*}

We write an empirical formular by fitting the experimental results in Fig. 2a and c as


(3) \begin{eqnarray*}{M}_1(B) = {M}_{1,\max }\left( {1 - {e}^{ - B/{B}_0}} \right),\end{eqnarray*}

where *M*_1_,_max_ is the maximum achievable value and *B*_0_ is defined as the characteristic magnetic field intensity determined by the sample properties (magnetic susceptibility, aspect ratio of flakes, etc.). *M*_2_ is given by *M*_2_ = *M* − *M*_1_, where *M* is the total number of effective layers (see Supplementary data). By fitting the data in Fig. 2a and c and Fig. S3, we obtain, for VSe_2_ suspension, *M*_1_,_max_ = 93 and *B*_0_ = 200 mT, and for graphene suspension, *M*_1_,_max_ = 13.4 and *B*_0_ = 115 mT. Consequently, the results in Fig. 2 are well-fitted by using Equations (1–3) (Fig. S3).

Overall, magnetic-field-driven flake alignment amplifies the in-plane non-linear response and thus enhances the total *χ*^(3)^, with the upper *χ*^(3)^ limit set by the intrinsic in-plane *χ*^(3)^ of fully ordered flakes. Ideal materials capable of achieving this limit require both large intrinsic in-plane *χ*^(3)^ and high magnetic susceptibility. Morphologically, high-performance flakes demand large lateral dimensions to suppress edge scattering and superior planar flatness, ensuring the entire *ab*-plane aligns parallel to the optical field.

### Magnetic-field-controlled all-optical switch based on SSPM

We demonstrate all-optical switch based on magnetic-field-controlled SSPM. The experimental set-up is schematically depicted in Fig. 4a (see descriptions in Materials and methods section). Two continuous-wave laser beams are employed. The first beam has a wavelength of *λ*_1_ = 532 nm, with intensity *I*_1_ and polarization *θ*_1_. The second beam has a wavelength of *λ*_2_ = 589 nm, with intensity *I*_2_ and polarization *θ*_2_. The two laser beams propagate with a small angle (<2°) and spatially overlap within a VSe_2_ suspension. Such all-optical switch based on SSPM (without an external field) had been initially proposed and realized in Ref. [1]. Herein, the patterns of the two laser beams both exhibit an identical number of rings *N*. The change in the intensity of one beam affects the SSPM pattern of the other beam, forming an all-optical switch.

**Figure 4.**
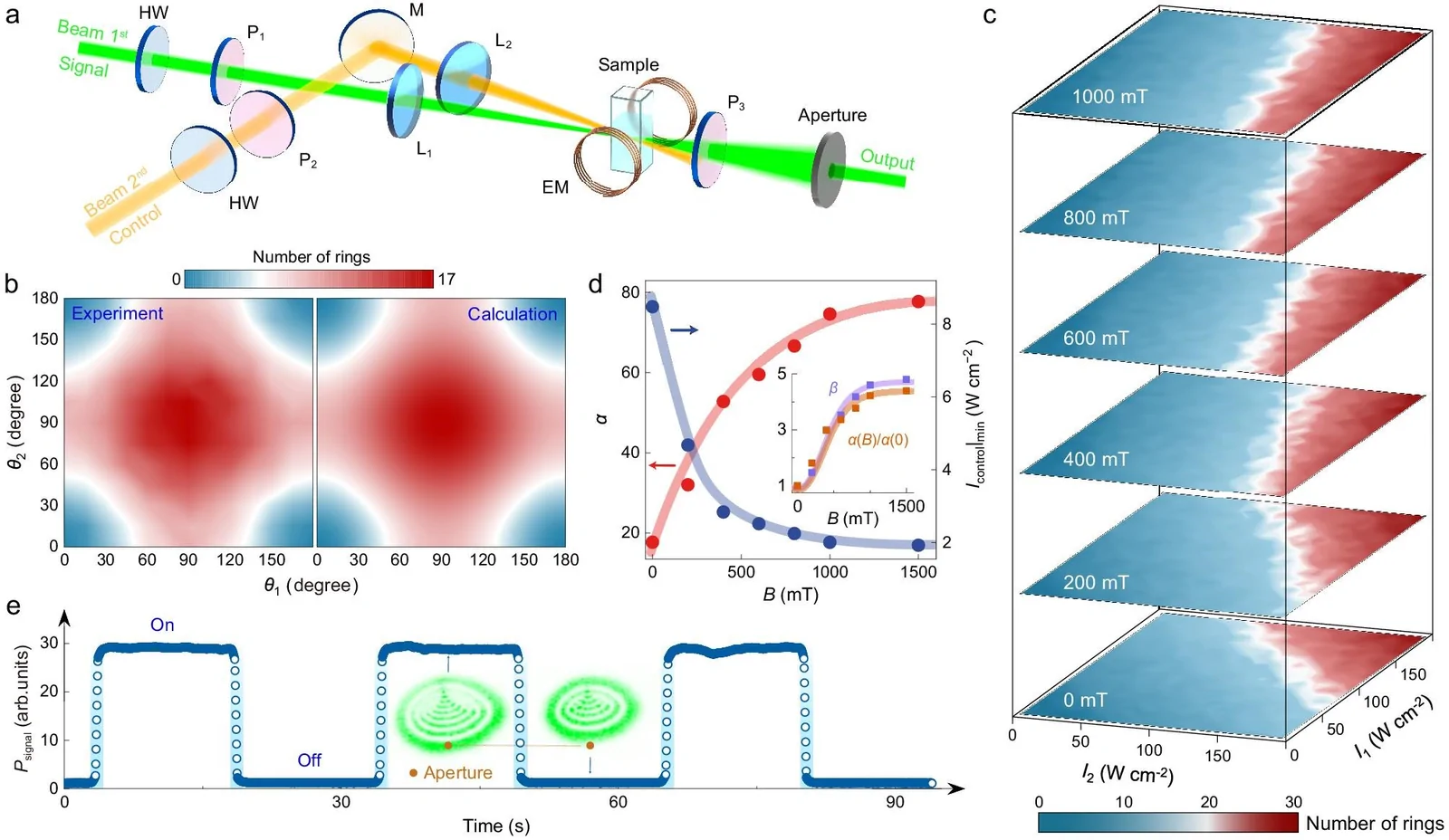
Magnetic-field-enhanced all-optical switch based on SSPM in VSe_2_. (a) Schematic experimental set-up of magnetic-field-enhanced all-optical switch. M: mirror. (b) Polarization dependence of the switch: *N* depending on *θ*_1_ and *θ*_2_. The calculated result is consistent with experimental data. (c) Magnetic field control of the switch: *N*–*I* relations under different *B* field intensities. White stripes: revealing the magnetic-field-controlled anisotropic factor *β*. (d) Enhanced performance of the switch: signal-to-control intensity ratio *α* (red dots) and lowest operating intensity *I*_control_|_min_ (blue dots). Inset: the *α* enhancement *α*(*B*)/*α*(0) is consistent with *β*. The curve for *β* is calculated by using Equation (4). (e) Real-time sequence of the switching operation. Insets: SSPM patterns of the signal beam in its ‘On’ and ‘Off’ states. An aperture is located in real space to capture the bright and dark rings of the pattern.

Next, we investigate the *B*-field control of the SSPM all-optical switch. The laser polarization dependence of the all-optical switch is recorded. The values of *N* under different *θ*_1_ and *θ*_2_ under fixed *I*_1_ = *I*_2_ = 100 W/cm^2^ and *B* = 600 mT is summarized in Fig. 4b. A C_4_ rotational symmetry is clearly observed in the experiment, elucidating an equal and parallel role for both beams in magnetic-field-controlled all-optical switch. The value of *N* increases to reach a maximum when *θ*_1_ and *θ*_2_ approach 90°, reading *N* = 17. In conventional set-up with *B* = 0 T, it is known [1] that *N* ∝ (*I*_1_ + *I*_2_) and *N* is independent of *θ*_1_ and *θ*_2_. Intriguingly, with *B* ≠ 0 T, we observe unambiguous dependence of *N* on *θ*_1_ and *θ*_2_ (Fig. 4b). This new phenomenon can only be explained by the magnetic field control of the switch.

Theoretically, we derive $N = 2.4 \times {10}^4{\pi }^2L\chi _{}^{{\mathrm{(3)}}}({\lambda }_1,\ {\theta }_1)/{\lambda }_1c{n}_0( {{I}_1 + \beta {I}_2} )$ (see Supplementary data), where we define $\beta = \frac{{\chi _{}^{{\mathrm{(3)}}}({\lambda }_2,\ {\theta }_2)}}{{\chi _{}^{{\mathrm{(3)}}}({\lambda }_1,\ {\theta }_1)}}\frac{{{\lambda }_1}}{{{\lambda }_2}}$ as the anisotropic factor of *χ*^(3)^. Both the magnetic field and the material’s properties may determine the anisotropic factor. The calculation results are consistent with the experimental results (Fig. 4b). For *B* = 0 mT, *χ*^(3)^ (*θ*) is isotropic; thus, *β* = 1 and the *N*–*I* relation is consistent with the conventional [1] *N* ∝ (*I*_1_ + *I*_2_) result (given *λ*_1_ is close to *λ*_2_). For non-zero *B* field intensity, given that *χ*^(3)^ (*θ*) reaches a maximum value $\chi _ \bot ^{(3)}$ at *θ* = 90° and a minimum value $\chi _\parallel ^{(3)}$ at *θ* = 0° (Fig. 2a), *β* assumes a maximum value at *θ*_1_ = 0° and *θ*_2_ = 90°, reading *β*_max_ = $\chi _ \bot ^{(3)}{\lambda }_1/\chi _\parallel ^{(3)}{\lambda }_2$.

For the magnetic field control of the switch, we record the *N*–*I* relations under different *B* field intensities. With fixed *θ*_1_ = 0° and *θ*_2_ = 90° as mentioned above, the *N*–*I* relations are presented in Fig. 4c. *N* increases linearly with *I*_1_ and *I*_2_. For clarity, the *N* = 20 results are marked in white, forming white contour bands. The white stripe rotates clockwise as *B* increases, manifesting the magnetic field control of the switch performance. The value of *β* can be directly obtained from the slopes *k* of white band in Fig. 4c, which is derived as *β* = −*k* (see Supplementary data). As presented in Fig. 4d, such magnetic field control yields enhanced values of *β*, from *β* = 1 at 0 mT to *β* = 4.8 at 1500 mT. Given that $\chi _{{\mathrm{one - layer}}}^{{\mathrm{(3)}}}({\lambda }_1)$ = 4.30 × 10^−9^ e.s.u. and $\chi _{{\mathrm{one - layer}}}^{{\mathrm{(3)}}}({\lambda }_2)$ = 4.25 × 10^−9^ e.s.u. (Fig. 1e), which are very close to each other, from Equation (1), the *B*-dependence of *β* is written as


(4) \begin{eqnarray*}\beta ( B) = \frac{{{M}_1{{(B)}}^2 + {M}_2{{(B)}}^2}}{{{M}_2{{(B)}}^2}}\frac{{{\lambda }_1}}{{{\lambda }_2}}.\end{eqnarray*}

Substituting the empirical formular Equation (3) into Equation (4), we obtain the quantitative dependence of *β* on *B*, which is very much consistent with our experimental results (Fig. 4d).

We further elucidate that anisotropic factor *β* is exactly the gain factor of the signal-to-control intensity ratio of the all-optical switch. In Fig. 4a, the switching is achieved by employing an aperture in front of the photo-receiver in the far field. Setting the first beam as the signal beam, with *I*_signal_ = 150 W/cm^2^ and *θ*_signal_ = 0°, and the second beam as the control beam, with tunable *I*_control_ and *θ*_control_ = 90°, the strong signal beam is controlled by the weak control beam. To achieve the optimized weak–control–strong capability, the minimum intensity of *I*_control_ is significant. The value of lowest operational *I*_control_, denoted by *I*_control_|_min_, is obtained by gradually increasing *I*_control_ until the outermost ring at the aperture location turns into a dark ring, as tuned by the control beam (inset of Fig. 4e). Interestingly, for identical *I*_signal_, the value of *I*_control_|_min_ decreases with increasing *B* field intensity, from 8.48 W/cm^2^ at 0 mT to 1.93 W/cm^2^ at 1500 mT.

The signal-to-control intensity ratio *α* = *I*_signal_/*I*_control_ maximizes at *I*_control_|_min_. Consequently, *α* is significantly enhanced by the magnetic field (Fig. 4d), from *α* = 17.7 at *B* = 0 mT to *α* = 77.7 at *B* = 1500 mT. To quantify the enhancement in *α*, we plot *α*(*B*)/*α*(0) in the inset of Fig. 4d. We also plot the field-dependence of *β* in the same inset. The two curves look very similar in all aspects, which indicates that the magnetic-field-induced anisotropic factor *β* is the driving force of the enhancement in *α*. Furthermore, we modulate the control beam using a square wave to obtain the real functioning temporal sequence of this switch, while the signal beam keeps a constant input power. The power of the output signal beam exhibits a magnetic-field-controlled square wave (Fig. 4e), which demonstrates the first SSPM all-optical switch controlled by external magnetic field.

## CONCLUSION

In summary, we discover magnetic-field-controlled SSPM for the first time. Owing to this, an unprecedented highest value of *χ*^(3)^ is achieved, exceeding 32% of the record obtained 14 years ago [6,33]. Significantly, magnetic-field-induced anisotropic SSPM and optical birefringence in SSPM are observed, which have not been reported before, enabling a new controlling method for SSPM by simply tuning the laser polarization. Both phenomena can be explained by a microscopic flake alignment picture, and the latter further supports the laser-induced electron coherence mechanism. Furthermore, we demonstrate a magnetic-field-controlled and magnetic-field-enhanced all-optical switch, which achieves 340% enhancement in the signal-to-control intensity ratio. Magnetic-field-controlled SSPM is universally applicable for extensive 2D magnetic quantum materials [35–38]. Our results shine light on magnetic-field-controlled coherent non-linear optical response in quantum materials, enabling broad applications in non-linear photonics devices based on various 2D magnetic materials [35–38].

## MATERIALS AND METHODS

### Preparation of the dispersive suspensions

The dispersive suspension of VSe_2_ is freshly prepared before experiments. First, 8 mg VSe_2_ flakes are suspended in 8 mL *N*-methyl-2-pyrrolidinone (NMP) solvent and sonicated for 30 min. The supernate is transferred into a tube, replenished with NMP upto 8 mL in total. Then, the tube with the suspension is centrifuged at 2500 rpm for 10 min. We collect the supernate and replenish it upto 8 mL again, obtaining the final suspension. A portion of the suspension is then transferred into a cuvette for the experiments.

To determine the concentration of the sample, we collect the precipitate in the aforementioned sonication and centrifugation processes. Residual NMP in the precipitate is removed using ethanol. After drying, we obtain 7.6 mg VSe_2_ flakes. Thus, the final suspension consists of 8 mL NMP and 0.4 mg VSe_2_, from which we obtain the concentration of 0.05 g L^−1^, or *ρ* = 2.39 × 10^−4^ mol L^−1^ in mole concentration (given the relative molecular mass of VSe_2_ as 209 g mol^−1^). The purpose of removing >95% of the VSe_2_ flakes is to ensure that most flakes in the suspension are thin enough to exhibit ferromagnetism [29].

The dispersive suspension of graphene is freshly prepared before experiments. We suspend 2 mg few-layer graphene flakes in 70 mL NMP (i.e., 0.0026 g L^−1^). After sonication, no prominent precipitation is observed. We take 2 mL suspension and dilute it into 4 mL, obtaining a final suspension with a concentration of 0.0013 g L^−1^ and *ρ* = 5.36 × 10^−5^ mol L^−1^.

### Experimental set-up for magnetic-field-controlled SSPM

The experimental set-up is schematically illustrated in Fig. 1a. Continuous-wave laser beams with different wavelengths are employed. The laser intensity and polarization are controlled using a half-wave plate and a polarizer. A convex lens with 200 mm focal length focuses the laser beam onto a suspension contained in a 10-mm-thick cuvette. The lens-to-cuvette distance is 150 mm. An external magnetic field is applied to the suspension, perpendicular to the laser beam propagation direction. The field is generated by an electromagnet and measured by a Gauss meter. During the experiment, SSPM diffraction patterns emerge on a white screen placed 3 m away.

### Measuring Δ*n*_eo_ from the polarization distribution of transmitted light

We set the polarization of the incident beam to be *θ* = 45°. Denoting the electric field as ${E}_{{{\rm incident}}} = {E}_0{e}^{i( {\omega t\!-\!{{{\check{k}}}}\cdot\!{{\check{r}}}} )}$, the electric fields for the ordinary and extraordinary lights are ${E}_{{\mathrm{incident}}}\cos ( {{{45}}^\circ } )$ and ${E}_{{\mathrm{incident}}}\sin ( {{{45}}^\circ } )$, respectively, both being ${E}_0{e}^{i( {\omega t \!-\! {\mathrm{{\check{k}}}} \cdot\! {\mathrm{\check{r}}}} )}/\sqrt{2} $. After traversing the suspension, the electric field becomes ${E}_{\mathrm{o}} = {E}_0{e}^{i( {\omega t \!-\! {\mathrm{{\check{k}}}} \cdot\! {\mathrm{\check{r}}} + 2\pi {n}_{\mathrm{o}}L/\lambda } )}/\sqrt{2} $ for ordinary light and ${E}_{\mathrm{e}} = {E}_0{e}^{i( {\omega t \!-\! {\mathrm{{\check{k}}}} \cdot\! {\mathrm{\check{r}}} + 2\pi {n}_{\mathrm{e}}L/\lambda } )}/\sqrt{2} $ for extraordinary light. Thus, the electric field after the polarizer is ${E}_{{\mathrm{out}}} = {E}_{\mathrm{o}}\cos ( \Phi ) + {E}_{\mathrm{e}}\sin ( \Phi )$, yielding


(5) \begin{eqnarray*}
{P}_{{\mathrm{out}}} &=& \frac{1}{2}{\varepsilon }_0{\big| {{E}_{{\mathrm{out}}}} \big|}^2\\
& =& {P}_0\big[ {1 + \cos \big( {{\mathrm{2}}\pi {\mathrm{\Delta }}{n}_{{\mathrm{eo}}}L/\lambda } \big)}\, {\sin \big( {2\Phi } \big)} \big],
\end{eqnarray*}

where ${P}_0 = {\varepsilon }_0E_0^2/2$ is the incident laser power and Δ*n*_eo_ = *n*_e_ − *n*_o_ is the refractive index difference. In our experiment, *P*_0_ = 30 mW and *λ* = 532 nm. By fitting the data in Fig. 3a and b using Equation (5), we obtain the values of Δ*n*_eo_ in Fig. 3c.

## Supplementary Material

nwag594_Supplemental_File
