# Supplementary material for: Magnetic-field-enhanced laser-induced electron coherence in 2D magnetic materials
Source: Natl Sci Rev. 2026 Sep 12;13(20):nwag594. doi: 10.1093/nsr/nwag594 (PMC13702035; doi:10.1093/nsr/nwag594)
Supplement: Supplementary file 1 [file nwag594_supplemental_file.docx]

Supplemental Material for

**Magnetic-field-enhanced laser-induced electron coherence in 2D magnetic materials**

Yixuan Huang,^1,2^ Sheng Meng,^1,2,3^ and Jimin Zhao^1,2,3,^*

^1^ *Beijing National Laboratory for Condensed Matter Physics, Institute of Physics, Chinese Academy of Sciences, Beijing 100190, China*

^2^ *School of Physical Sciences, University of Chinese Academy of Sciences, Beijing 100049, China*

^3^ *Songshan Lake Materials Laboratory, Dongguan, Guangdong 523808, China*

*** Corresponding authors. E-mails: jmzhao@iphy.ac.cn

**CONTENTS**

Supplementary Text

Figs. S1 to S7

Table S1

**I. Extracting ring number *N* from the SSPM pattern**

The values of *N* are obtained by manually counting the bright and dark fringes in the SSPM pattern. As illustrated in Fig. S4, the counting starts from the outermost fringe, which corresponds to zero phase shift and is always bright, assigned as zero order. From the edge to the center, each bright or dark fringe contributes 0.5 to *N*, because the phase difference increases by half a period (i.e., *π*) between adjacent fringes. Therefore, the uncertainty in *N* is estimated to be ±0.5, because *N* is an integer multiple of 0.5.

If the fringe contrast becomes too weak for direct counting (did not occur in our experiments), *N* can be obtained from the diameter *D* of SSPM pattern using the linear *N*-*D* relation, derived as follows:

The half-cone angle of the outcoming laser beam is, where is the nonlinear optical phase and *r* is the radial coordinate of the laser beam. Hence, we have, which reaches its maximum value at $r=\pm r_{0}/\text{ }2$. According to, we obtain $\beta=\frac{2\lambda}{e^{1/2}r_{0}}N$. For SSPM pattern projected onto a screen at a distance *Z* from the sample, the diameter is

$D=2Z\tan\left(\beta\right)=2Z\tan\left(\frac{2\lambda}{e^{1/2}r_{0}}N\right)$.

For small *β*,

$D=2Z\beta=\frac{4\lambda Z}{e^{1/2}r_{0}}N$.

In conclusion, *N* can be obtained indirectly by measuring *D*.

**II. Measuring *χ*^(3)^ from the *N*-*I* relations in SSPM**

In SSPM, *n* = *n*_0_ + *n*_2_*I*, where *n*, *n*_0_ and *n*_2_ are the total, linear, and nonlinear refractive indices. The acquired phase shift *Δφ* for a laser beam after traversing a sample with a thickness *L* is, where *r* is the radial coordinate and *λ* is the laser wavelength in vacuum^15^. For a gaussian laser beam with average intensity *I*, the laser intensity in the center and edge is 2*I* and 0, respectively, corresponding to a phase difference of. *N* is thus obtained by. Given $n_{2}=1.2\times10^{4}\pi^{2}\chi^{(3)}/n_{0}^{2}c$, the overall nonlinear coefficient^2,13^ is $\chi_{\text{total}}^{\text{(3)}}=\frac{c\lambda n_{0}}{2.4\times10^{4}\pi^{2}L}\times\frac{N}{I}$. Because the *N*-*I* relation is linear, passing through the (0,0) point, *N*_max_/*I*_max_ = *dN*/*dI*. Thus, $\chi_{\text{total}}^{\text{(3)}}$ can be easily obtained through the slope of the *N*-*I* relation $\chi_{\text{total}}^{\text{(3)}}=\frac{c\lambda n_{0}}{2.4\times10^{4}\pi^{2}L}\times\frac{dN}{dI}$. Next, the effective one-layer nonlinear coefficient $\chi_{\text{one-layer}}^{\text{(3)}}$ is obtained by^2,13^ $\chi_{\text{total}}^{\text{(3)}} = M^{2}\chi_{\text{one-layer}}^{\text{(3)}}$, where *M* is the effective number of layers (Methods).

Regarding the limitation of obtaining $\chi_{\text{one-layer}}^{\text{(3)}}$, the experiment needs to be performed under appropriate conditions. For example, the laser intensity needs to be in the linear regime of the *N*-*I* relations, not being too high or too low [6]; the flake concentration needs to be low enough to prevent precipitation; the penetration depth needs to be considered for high-absorbing samples [16].

**III. Polarization dependence of *χ*^(3)^**

We denote the laser beam propagation direction as *z*-axis and the magnetic field direction as *x*-axis. For the *i*th effective layer, we denote Ω*_i_* as the angle between the *c*-axis and *B* field (*x*-axis), and *α_i_* as the azimuth angle. The *c*-axis is written as $\widehat{c_{i}}=\left(\begin{matrix} \cos(\Omega_{i}) & \sin(\Omega_{i})\cos(\alpha_{i}) & -\sin(\Omega_{i})\sin(\alpha_{i}) \end{matrix}\right)$. The electric field for laser beam with polarization *θ* is $\overrightarrow{E}=\left(\begin{matrix} E_{0}\cos(\theta) & E_{0}\sin(\theta) & 0 \end{matrix}\right)$. Thus, the in-plane electric field is $\overrightarrow{E_{ab,i}}=\sqrt{\left|\overrightarrow{E}\right|^{2}-\left(\overrightarrow{E}\cdot\widehat{c_{i}}\right)^{2}}$, corresponding to $I_{ab,i}\left(\theta\text{,Ω}_{i}\text{,}\alpha_{i}\right)$ $=I\left[1-\left(\cos(\text{Ω}_{i})\cos(\theta)\right.\right.$ $\left.\left.+\sin(\text{Ω}_{i})\cos(\alpha_{i})\sin(\theta)\right)^{2}\right]$. The acquired extra phase is, where $\Delta L=L/M$ is the distance between two effective layers. Given $n_{2}=1.2\times10^{4}\pi^{2}\chi_{\text{total}}^{(3)}/n_{0}^{2}c$ and $\chi_{\text{total}}^{\text{(3)}} = M^{2}\chi_{\text{one-layer}}^{\text{(3)}}$ (Section I of Supplementary Information),.

Consider the flake alignment in Figs. 3e and 3f:

(1) In Fig. 3e where Ω*_i_* = 0^°^ for ferromagnetic flakes, $I_{ab,i}=I\sin^{2}(\theta)$ and thus. The overall extra phase is $=4.8\times10^{4}\pi^{3}M^{2}\chi_{\text{one-layer}}^{\text{(3)}}LI\sin^{2}(\theta)/n_{0}\lambda c$. Given (Section I of Supplementary Information),

$\chi_{\text{total}}^{\text{(3)}}=M^{2}\sin^{2}(\theta)\chi_{\text{one-layer}}^{\text{(3)}}$.

(2) In Fig. 3e, where Ω*_i_* = 90^°^ for diamagnetic flakes, $I_{ab,i}=I\left[1-\cos^{2}(\alpha_{i})\sin^{2}(\theta)\right]$ and thus. Because *α_i_* is randomly distributed in [0, 2π], the overall extra phase is, where $\sum_{i=1}^{M} \Delta L\cos^{2}(\alpha_{i})=\frac{M\Delta L}{2\pi}\oint_{\alpha} \cos^{2}\left(\alpha\right)d\alpha=L\left(1-\frac{1}{2}\sin^{2}\left(\theta\right)\right)$. Similarly, we derive

$\chi_{\text{total}}^{\text{(3)}}=M^{2}\left(1-\frac{1}{2}\sin^{2}\left(\theta\right)\right)\chi_{\text{one-layer}}^{\text{(3)}}$.

However, not all flakes are aligned. For non-aligned flakes, $\chi_{\text{total}}^{\text{(3)}}$ is isotropic^2,13,16-27^ with $\left.\chi_{\text{total}}^{\text{(3)}}\right|_{\text{non-aligned}}=M^{2}\chi_{\text{one-layer}}^{\text{(3)}}$ (inset of Fig. 2e). Because, $\chi_{\text{total}}^{\text{(3)}}(\theta)=\left.\chi_{\text{total}}^{\text{(3)}}(\theta)\right|_{\text{aligned}}+\left.\chi_{\text{total}}^{\text{(3)}}(\theta)\right|_{\text{non-aligned}}$. Thus, for VSe_2_ suspension, from eq. (S1), we have

$\chi_{\text{total}}^{\text{(3)}}(\theta)=\left(M_{1}^{2}\sin^{2}(\theta)+M_{2}^{2}\right)\chi_{\text{one-layer}}^{\text{(3)}}$,

which is exactly eq. (1); and for graphene suspension, from eq. (S2), we have

$\chi_{\text{total}}^{\text{(3)}}(\theta)=\left(M_{1}^{2}\left(1-\frac{1}{2}\sin^{2}\left(\theta\right)\right)+M_{2}^{2}\right)\chi_{\text{one-layer}}^{\text{(3)}}$,

which is exactly eq. (2).

**IV. Determine the ordinary and extraordinary lights in the sample**

Denoting the laser beam propagation direction as *z*-axis and the magnetic field direction as *x*-axis, the dielectric constant of the sample is written as

$\widehat{\varepsilon}=\left(\begin{matrix} \varepsilon_{x} & & \\ & \varepsilon_{y} & \\ & & \varepsilon_{z} \end{matrix}\right)$.

The suspension has a rotational symmetry along the magnetic field (*x*-axis). Thus, mathematically the suspension preserves an identical dielectric constant tensor upon a rotation along the *x*-axis. Given the rotation matrix along the *x*-axis as

,

where *ϕ* is the rotating angle, the dielectric constant after rotation is

.

Enforcing $\widehat{\varepsilon}'=\widehat{\varepsilon}$ for an arbitrary angle *ϕ*, the only valid solution is $\varepsilon_{y}=\varepsilon_{z}$. Thus,

$\widehat{\varepsilon}=\left(\begin{matrix} \varepsilon_{x} & & \\ & \varepsilon_{y} & \\ & & \varepsilon_{y} \end{matrix}\right)$.

Similarly, the magnetic permeability $\mu_{y}=\mu_{z}$. Consequently, we have the refractive index $n_{y}=n_{z}$ because $n=\sqrt{\mu\varepsilon/\mu_{0}\varepsilon_{0}}$. Therefore, supposing there is a laser beam propagating along the *x*-axis (polarized within the *y-z* plane), we expect it experience no optical birefringence. In such a way, we could determine the *x*-axis is the optical axis in the optical birefringence. For the experiments in the main text, the ordinary light polarizes along the *x*-axis and the extraordinary light polarizes perpendicularly (inset of Fig. 3a).

**V. Calculation of the number of effective layers**

We estimate the number of effective layers *M* based on Ref. [2]. For the VSe_2_ sample, the total number of VSe_2_ primitive cells in the cuvette is *M*_cell_ = *ρ*×*V*×*N*_A_ = 5.76×10^17^, where *V*=1×1×4 cm^3^ is the volume of cuvette and *N*_A_ is the Avogadro constant. From the lattice constants [44] *a* = *b* = 3.34 Å and *c* = 6.12 Å for VSe_2_, the unit cell area in its *ab*-plane is *S*_cell__*_ab_* = (3.34 Å)^2^×sin 60^°^= 9.66×10^-16^ cm^2^. Given the lateral area of the cuvette *S*_lateral_=1×4 cm^2^, the number of primitive cells tiling up to cover one effective layer is *m* = *S*_lateral_/*S*_cell__*_ab_* = 4.14×10^15^. Hence the number of VSe_2_ effective layers that the laser beam traverses is *M* = *M*_cell_/*m* = 139.

Similarly, for the graphene sample, we have *M*_cell_ = *ρ*×*V*×*N*_A_ = 1.29×10^17^ and *S*_cell__*_ab_* = (1.422 Å×2×cos 30^°^)^2^×sin 60^°^= 5.25×10^-16^ cm^2^ (given the carbon-carbon distance [45] of 1.422 Å). Thus, *m* = *S*_lateral_/*S*_cell__*_ab_* = 7.62×10^15^ and *M* = *M*_cell_/*m* = 17.

**VI. *N*-*I* relation in magnetic-field-controlled all-optical switch**

The phase shifts contributed by the beam 1^st^ and beam 2^nd^ are and, respectively. The total phase shift is. With $n_{2}=1.2\times10^{4}\pi^{2}\chi^{(3)}/n_{0}^{2}c$ and, we obtain

$N=2.4\times10^{4}\pi^{2}L\left(\chi^{\text{(3)}}(\lambda_{1},\text{ }\theta_{1})I_{1}/\lambda_{1}+\chi^{\text{(3)}}(\lambda_{2},\text{ }\theta_{2})I_{2}/\lambda_{2}\right)/cn_{0}$.

We have $N=2.4\times10^{4}\pi^{2}L\chi^{\text{(3)}}(\lambda_{1},\text{ }\theta_{1})/\lambda_{1}cn_{0}\left(I_{1}+\beta I_{2}\right)$, where *β* is the anisotropic factor (Maintext) controlled by the *B* field intensity.

**VII. Obtaining the value of anisotropic factor *β***

From the aforementioned $N=2.4\times10^{4}\pi^{2}L\chi^{\text{(3)}}(\lambda_{1},\text{ }\theta_{1})/\lambda_{1}cn_{0}\left(I_{1}+\beta I_{2}\right)$, the derivatives $\left(\frac{\partial N}{\partial I_{1}}\right)_{I_{2}}=C$ and $\left(\frac{\partial N}{\partial I_{2}}\right)_{I_{1}}=\beta C=\beta\left(\frac{\partial N}{\partial I_{1}}\right)_{I_{2}}$， where $C\equiv2.4\times10^{4}\pi^{2}L\chi^{\text{(3)}}(\lambda_{1},\text{ }\theta_{1})/\lambda_{1}cn_{0}$. Thus, *β* can be obtained by $\beta=\left(\frac{\partial N}{\partial I_{2}}\right)_{I_{1}}\left(\frac{\partial N}{\partial I_{1}}\right)_{I_{2}}^{-1}=\left(\frac{\partial N}{\partial I_{2}}\right)_{I_{1}}\left(\frac{\partial I_{1}}{\partial N}\right)_{I_{2}}$. The slope *k* of the white band in Fig. 4(c) is $k=\left(\frac{\partial I_{1}}{\partial I_{2}}\right)_{N}$. From the cyclic relation of partial derivatives, $\left(\frac{\partial N}{\partial I_{2}}\right)_{I_{1}}\left(\frac{\partial I_{1}}{\partial N}\right)_{I_{2}}\left(\frac{\partial I_{2}}{\partial I_{1}}\right)_{N}=-1$, we derive $\left(\frac{\partial N}{\partial I_{2}}\right)_{I_{1}}\left(\frac{\partial I_{1}}{\partial N}\right)_{I_{2}}=-\left(\frac{\partial I_{1}}{\partial I_{2}}\right)_{N}$. Consequently, *β* can be experimentally measured by recording the slope *k* using $\beta=-k$.

**VIII. Viscosity effect of magnetic-field-controlled SSPM**

As a further verification of magnetic-field-induced flake orientation, we investigate magnetic-field-controlled optical birefringence in suspension of different viscosities. The viscosity is varied by adding different amount of water to NMP solution. The viscosity *η* of the NMP-water solution non-monotonically depends on NMP mole fraction *x*_NMP_ (Fig. S5b)^[R1]^. During the experiment, a laser beam with a wavelength of 532 nm and a power of 0.8 mW continuously illuminates the sample. Two polarizers, with their transmission axes oriented at *θ* = 45^°^ and *θ* = 135^°^, are placed before and after the sample, respectively. The transmitted power after the latter polarizer indicates the intensity of optical birefringence.

Initially, no magnetic field is applied. Upon the abrupt application of a magnetic field, we record the evolution of the transmitted power. The experiments repeat under different *x*_NMP_, we measured the evolution of optical birefringence (Fig. S5a) and obtain the 1/*e* characteristic time *τ*. Figure S5b compares the *τ*-*x*_NMP_ dependence with the *η*-*x*_NMP_ dependence, showing their high consistency. Figure S5c further demonstrates the positive dependence of *τ* on *η*, which indicates that the flake orientation is retarded by the liquid. Therefore, our results support the flake orientation mechanism.

**IX. Anisotropic scatterings of graphene and VSe_2_ suspensions under magnetic field**

To verify the flake alignment, we measure the 10^°^ forward scattering intensity for both graphene and VSe_2_ suspensions (Fig. S6). The measurement is performed under incident laser polarizations of *θ* = 0^°^ and *θ* = 90^°^, respectively. The incident laser intensity is 1 mW and the laser wavelength is 532 nm.

For graphene (Fig. S6a), without magnetic field, the scattering intensities *I*_scatter_ are identical for *θ* = 0^°^ and 90^°^. As *B* increases, the scattering intensity for *θ* = 0^°^ case enhances, while that for *θ* = 90^°^ case decreases. An anisotropic factor of scattering, defined as |*I*_scatter_(90^°^)-*I*_scatter_(0^°^)|/ (*I*_scatter_(90^°^)+*I*_scatter_(0^°^)), is extracted, which increases with *B* (Fig. S6b).

The anisotropic scattering (Fig. S6a) is consistent with the proposed flake alignment (Fig. 3f): The diamagnetic graphene flakes tend to align such that their the *B* field passes through their *ab*-planes. Thus, for *θ* = 0^°^, the optical electric field is parallel to the flakes’ *ab*-plane, which couples more efficiently to flakes’ electrons and results in higher scattering intensity (Fig. S6a). For *θ* = 90^°^, the optical electric field is perpendicular to the *B* field, and the flakes’ *ab*-plane is fully random relative to the optical field, making light couples less efficiently to the flakes’ electrons and results in smaller scattering intensity (Fig. S6a). As *B* increases, the flakes are better aligned, enhancing the above anisotropy in light scattering.

Similar results and analysis can be applied to VSe_2_.

**X. Potential energy of graphene and VSe_2_ flake under a magnetic field**

The magnetic susceptibiltiy tensor of flakes is expressed as $\overrightarrow{\overrightarrow{\chi}}=\sum_{i,j=1}^{3} \chi_{ij}\widehat{e_{i}}\widehat{e_{j}}$, where $\widehat{e_{i}}$ is the unit vector denoting the *a*, *b*, *c* directions of the flake when *i* = 1, 2, 3, respectively. The magnetization of flake is $\overrightarrow{m}=\overrightarrow{\overrightarrow{\chi}}\cdot\overrightarrow{B}/\mu_{0}=\sum_{i,j=1}^{3} \chi_{ij}\widehat{e_{i}}\left(\widehat{e_{j}}\cdot\overrightarrow{B}\right)/\mu_{0}$ and the potential energy is $U=-\overrightarrow{m}\cdot\overrightarrow{B}=$ $-\sum_{i,j=1}^{3} \chi_{ij}\left(\widehat{e_{i}}\cdot\overrightarrow{B}\right)\left(\widehat{e_{j}}\cdot\overrightarrow{B}\right)/\mu_{0}$. Thus, the explicit numerical expression of *U* demands a complete $\overrightarrow{\overrightarrow{\chi}}$ tensor, which, to our knowledge, is absent so far.

It has been proposed that the magnetization of VSe_2_ and graphene thin flakes is primary along the *c*-axis (out-of-plane) [30,31]. Thus, we reasonable simplify the *U* expression by only considering the $\chi_{33}$ (out-of-plane) component. By denoting $\chi_{33}$ as $\chi_{M}$, we have $U=-\chi_{M}\left(\widehat{e_{3}}\cdot\overrightarrow{B}\right)\left(\widehat{e_{3}}\cdot\overrightarrow{B}\right)/\mu_{0}=-\chi_{M}B^{2}\text{cos}^{2}(\Omega)/\mu_{0}$, where Ω is the angle between the flakes’ *c*-axis and the magnetic field (Fig. 3d).

**Figures and tables**

**Fig. S1.** Measuring *χ*^(3)^ values for VSe_2_ suspension (Fig. 2a) from the linear *N*-*I* relations under different laser polarizations and *B* field intensities. Solid lines: fitting of the experimental data.

**Fig. S2.** Measuring *χ*^(3)^ values for graphene suspension (Fig. 2c) from the linear *N*-*I* relations under different laser polarizations and *B* field intensities. Solid lines: fitting of the experimental data.

**Fig. S3.** Empirical discription of magnetic-field-controlled SSPM. **a,d,** Obtaining the explicit empirical fomular for VSe_2_ and graphene suspensions by fitting the experimental data in Fig. 2a, 2b. **b,c,** Calculated results (curves) for VSe_2_ suspension using eq. (1). **e,f,** Calculated results (curves) for graphene suspension using eq. (2). The data (dots) in **b,c,e,f** are from Fig. 2.

**Fig. S4.** Counting the number of rings *N*. The counting starts from the outermost fringe to the inner fringe. The yellow and red dots mark the bright and dark fringes, respectively. From outside to inside (i.e., from left to right), the orders of each dotted fringes are 0.5, 1, 1.5, 2, ... and 8. Thus, we have *N*=8 for this SSPM pattern.

**Fig. S5.** Viscosity effect of flake alignment probing by optical birefringence. **a,** Evolution of transmitted power in graphene samples with different NMP mole fraction *x*_NMP_, corresponding to different viscosity coefficients *η*. **b,** Comparison between 1/*e* characteristic time *τ* and viscosity coefficient *η*. **c,** Positively linear *τ*-*η* dependence.

**Fig. S6.** Anisotropic scattering experiment to verify the flake alignment in graphene and VSe_2_ suspensions under a magnetic field. **a,** Scattering intensity and **b,** anisotropic factor of the graphene suspension depending on *B*. **c,** Scattering intensity and **d,** anisotropic factor of the VSe_2_ suspension depending on *B*.

**Fig. S7.** Diagram of the investigation mind map.

**Table S1.** Absence of magnetic-field-controlled SSPM and optical birefringence in graphene-resin and VSe_2_-resin samples.

| *B* [mT] | graphene-resin | | VSe_2_-resin | |
| --- | --- | --- | --- | --- |
|  | *N* | *P*_out_/*P*_in_ | *N* | *P*_out_/*P*_in_ |
| 0 | 13 | 2.9×10^-3^ | 9 | 2.0×10^-3^ |
| 400 | 13 | 3.0×10^-3^ | 9 | 1.8×10^-3^ |
| 800 | 13 | 3.1×10^-3^ | 9 | 2.1×10^-3^ |
| 1200 | 13 | 3.1×10^-3^ | 9 | 2.3×10^-3^ |

Note: For SSPM measurements, the excitation laser beam has an intensity of 150 W/cm^2^, polarization of *θ* = 0^°^, and wavelength of 532 nm. The number of rings *N* is recorded. For optical birefringence measurements, the incident laser beam has a power of *P*_in_=1.2 mW and polarization of *θ* = 45^°^. A polarizer is placed after the sample with its transmission axis at *θ* = 135^°^, after which the transmitted power *P*_out_ is recorded. The results show *N* and *P*_135_ are not affected by the magnetic field.
